# Chlorido(pyridine-κ*N*)bis­[2-(quinolin-2-yl)phenyl-κ^2^ *C* ^1^,*N*]iridium(III) mono­hydrate

**DOI:** 10.1107/S1600536808025452

**Published:** 2008-08-30

**Authors:** Cheng Li, Xiao-Qing Dong, Quan Wang, Chun-Xia Ren, Yu-Qiang Ding

**Affiliations:** a School of Chemical and Materials Engineering, Jiangnan University, 1800 Lihu Road, Wuxi, Jiangsu, People’s Republic of China

## Abstract

In the neutral mononuclear iridium(III) title complex, [Ir(C_15_H_10_N)_2_Cl(C_5_H_5_N)]·H_2_O, the Ir atom is coordinated by two N atoms and two C atoms from two 2-(quinolin-2-yl)­phenyl ligands, one N atom from a pyridine ligand and one Cl atom in an octa­hedral geometry.

## Related literature

For related literature, see: Adachi *et al.* (2000 ▶); Baldo *et al.* (1998 ▶); Gao *et al.* (2002 ▶); Lamansky *et al.* (2001*a* ▶,*b* ▶); Liu *et al.* (2007 ▶).
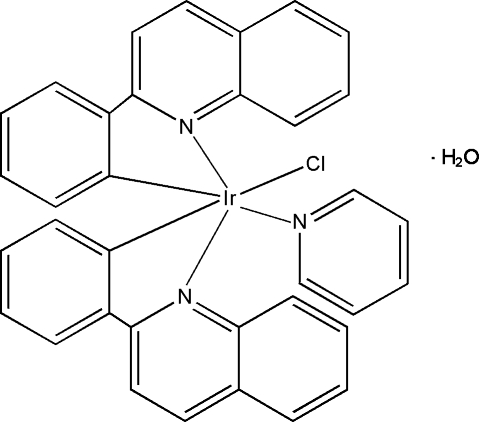


## Experimental

### 

#### Crystal data

[Ir(C_15_H_10_N)_2_Cl(C_5_H_5_N)]·H_2_O
*M* *_r_* = 733.25
Monoclinic, 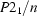

*a* = 9.8949 (15) Å
*b* = 17.653 (3) Å
*c* = 16.424 (3) Å
β = 98.545 (3)°
*V* = 2837.0 (8) Å^3^
*Z* = 4
Mo *K*α radiation
μ = 4.83 mm^−1^
*T* = 273 (2) K
0.16 × 0.12 × 0.08 mm

#### Data collection

Bruker SMART APEXII CCD area-detector diffractometer
Absorption correction: multi-scan (*SADABS*; Sheldrick, 1996 ▶) *T*_min_ = 0.511, *T*_max_ = 0.684
14857 measured reflections
5027 independent reflections
3702 reflections with *I* > 2σ(*I*)
*R*_int_ = 0.049

#### Refinement

*R*[*F* ^2^ > 2σ(*F* ^2^)] = 0.034
*wR*(*F* ^2^) = 0.075
*S* = 1.02
5027 reflections
370 parameters
9 restraints
H-atom parameters constrained
Δρ_max_ = 0.78 e Å^−3^
Δρ_min_ = −0.72 e Å^−3^

### 

Data collection: *APEX2* (Bruker, 2007 ▶); cell refinement: *SAINT* (Bruker, 2007 ▶); data reduction: *SAINT*; program(s) used to solve structure: *SIR97* (Altomare *et al.*, 1999 ▶); program(s) used to refine structure: *SHELXL97* (Sheldrick, 2008 ▶); molecular graphics: *PLATON* (Spek, 2003 ▶); software used to prepare material for publication: *SHELXL97*.

## Supplementary Material

Crystal structure: contains datablocks global, I. DOI: 10.1107/S1600536808025452/hy2145sup1.cif

Structure factors: contains datablocks I. DOI: 10.1107/S1600536808025452/hy2145Isup2.hkl

Additional supplementary materials:  crystallographic information; 3D view; checkCIF report

## Figures and Tables

**Table:** 

Ir1—C11	1.990 (6)
Ir1—C26	1.992 (6)
Ir1—N1	2.090 (5)
Ir1—N2	2.092 (5)
Ir1—N3	2.221 (5)
Ir1—Cl1	2.5182 (16)

**Table:** 

C11—Ir1—C26	87.3 (2)
C11—Ir1—N1	80.0 (2)
C26—Ir1—N1	93.3 (2)
C11—Ir1—N2	94.9 (2)
C26—Ir1—N2	80.0 (2)
N1—Ir1—N2	171.7 (2)
C11—Ir1—N3	173.07 (19)
C26—Ir1—N3	87.8 (2)
N1—Ir1—N3	105.16 (18)
N2—Ir1—N3	79.48 (17)
C11—Ir1—Cl1	96.67 (17)
C26—Ir1—Cl1	174.02 (16)
N1—Ir1—Cl1	83.02 (14)
N2—Ir1—Cl1	104.10 (14)
N3—Ir1—Cl1	88.68 (13)

**Fig. 1.**
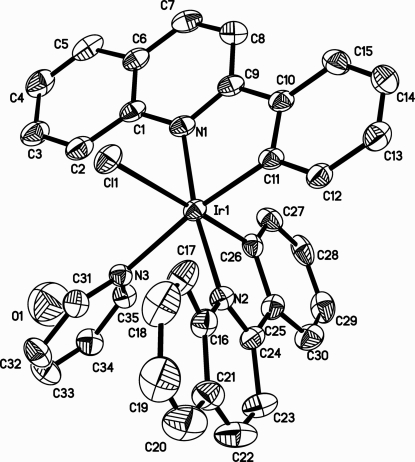
Molecular structure of the title compound. Displacement ellipsoids are drawn at the 50% probability level. H atoms have been omitted for clarity.

## supplementary crystallographic information

### Comment

Since the significant work by Thompson and Forrest (Adachi *et al.*, 2000; Baldo *et al.*, 1998), the chemistry of cyclometalated Ir^III^ complexes has received a great deal of attention. These homoleptic complexes, (CÑ)_2_Ir(LX), have proven to be very efficient when used in organic light emitting diodes (OLEDs), where CÑ is a general abbreviation used hereafter for a cyclometalating ligand and LX stands for other ligands. (CÑ)_2_Ir(LX) complexes with different ligands have various emissions (Gao *et al.*, 2002; Lamansky *et al.*, 2001*a*, b; Liu *et al.*, 2007).

In this paper, we report the crystal structure of the title compound, which is a neutral mononuclear complex. The Ir^III^ atom is coordinated by two N atoms and two C atoms from two 2-phenylquinoline (pq) ligands, one N atom from a pyridine ligand and one Cl atom in an octahedral geometry (Fig. 1). The Ir1—N1 and Ir1—N2 bond lengths are 2.090 (5) and 2.092 (5)Å (Table 1) and agree well with those observed in the related (CÑ)_2_Ir(LX) complexes (Gao *et al.*, 2002; Lamansky *et al.*, 2001*a*). The Ir—C bond lengths of 1.990 (6) and 1.992 (6)Å are slightly shorter than the Ir—C bond length [2.003 (9) Å] in the complex [Ir(ppy)_2_(acac)] (ppy = 2-pyridylphenyl; acac = acetylacetone) (Lamansky *et al.*, 2001*a*). The N—Ir—C angles of 80.0 (2)° are comparable to that [81.7 (4)°] in [Ir(ppy)_2_(acac)].

### Experimental

A mixture of (pq)_2_IrCl (0.126 g, 0.2 mmol) and sodium bicarbonate (0.04 g, 0.5 mmol) dissolved in pyridine (12 ml) and dichloromethane (10 ml) was refluxed for 24 h and then cooled to room temperature. The solvent was removed in vacuum. The residue was washed with hexane and hot water. The crude product was separated by chromatography on silica gel with dichloromethane as eluent to give a red solid. Single crystals suitable for X-ray diffraction were obtained by slow diffusion of hexane into the dichloromethane solution.

### Refinement

H atoms bonded on C atoms were positioned geometrically and refined as riding atoms, with C—H = 0.93 Å and *U*_iso_(H) = 1.2*U*_eq_(C). H atoms of water molecule can not be located in difference Fourier map and they were not included in refinements.

### Crystal data

**Table:**

[Ir(C_15_H_10_N)_2_Cl(C_5_H_5_N)]·H_2_O	*F*_000_ = 1440
*M**_r_* = 733.25	*D*_x_ = 1.717 Mg m^−^^3^
Monoclinic, *P*2_1_/*n*	Mo *K*α radiation λ = 0.71073 Å
Hall symbol: -P 2yn	Cell parameters from 3064 reflections
*a* = 9.8949 (15) Å	θ = 2.5–23.5º
*b* = 17.653 (3) Å	µ = 4.83 mm^−^^1^
*c* = 16.424 (3) Å	*T* = 273 (2) K
β = 98.545 (3)º	Block, red
*V* = 2837.0 (8) Å^3^	0.16 × 0.12 × 0.08 mm
*Z* = 4	

### Data collection

**Table:**

Bruker SMART APEXII CCD area-detector diffractometer	5027 independent reflections
Radiation source: fine-focus sealed tube	3702 reflections with *I* > 2σ(*I*)
Monochromator: graphite	*R*_int_ = 0.049
*T* = 273(2) K	θ_max_ = 25.1º
φ and ω scans	θ_min_ = 1.7º
Absorption correction: multi-scan(SADABS; Sheldrick, 1996)	*h* = −11→10
*T*_min_ = 0.511, *T*_max_ = 0.684	*k* = −20→21
14857 measured reflections	*l* = −16→19

### Refinement

**Table:**

Refinement on *F*^2^	Secondary atom site location: difference Fourier map
Least-squares matrix: full	Hydrogen site location: inferred from neighbouring sites
*R*[*F*^2^ > 2σ(*F*^2^)] = 0.034	H-atom parameters constrained
*wR*(*F*^2^) = 0.075	*w* = 1/[σ^2^(*F*_o_^2^) + (0.0276*P*)^2^ + 1.7551*P*] where *P* = (*F*_o_^2^ + 2*F*_c_^2^)/3
*S* = 1.02	(Δ/σ)_max_ = 0.001
5027 reflections	Δρ_max_ = 0.78 e Å^−^^3^
370 parameters	Δρ_min_ = −0.72 e Å^−^^3^
9 restraints	Extinction correction: none
Primary atom site location: structure-invariant direct methods	

### Fractional atomic coordinates and isotropic or equivalent isotropic displacement parameters (Å^2^)

**Table:**

	*x*	*y*	*z*	*U*_iso_*/*U*_eq_	
Ir1	0.09759 (3)	0.240147 (11)	0.005833 (14)	0.03528 (9)	
Cl1	0.20494 (18)	0.31961 (8)	−0.09466 (10)	0.0514 (4)	
N1	0.0132 (5)	0.3437 (2)	0.0339 (3)	0.0410 (13)	
N2	0.1566 (5)	0.1294 (3)	−0.0176 (3)	0.0394 (12)	
N3	0.2997 (5)	0.2349 (2)	0.0852 (3)	0.0382 (11)	
O1	0.4549 (12)	0.4379 (6)	0.9584 (8)	0.245 (5)	
C1	0.0782 (7)	0.4038 (3)	0.0794 (4)	0.0442 (17)	
C2	0.2205 (8)	0.4034 (3)	0.1040 (4)	0.056 (2)	
H2	0.2725	0.3631	0.0893	0.067*	
C3	0.2822 (8)	0.4615 (4)	0.1490 (5)	0.066 (2)	
H3	0.3764	0.4605	0.1648	0.080*	
C4	0.2069 (9)	0.5232 (4)	0.1724 (5)	0.067 (2)	
H4	0.2501	0.5617	0.2051	0.081*	
C5	0.0719 (9)	0.5258 (4)	0.1467 (4)	0.062 (2)	
H5	0.0226	0.5678	0.1598	0.074*	
C6	0.0025 (7)	0.4663 (3)	0.1002 (4)	0.0464 (17)	
C7	−0.1381 (8)	0.4686 (3)	0.0732 (4)	0.0566 (19)	
H7	−0.1897	0.5093	0.0874	0.068*	
C8	−0.1989 (8)	0.4119 (3)	0.0268 (4)	0.0541 (18)	
H8	−0.2924	0.4135	0.0084	0.065*	
C9	−0.1209 (7)	0.3500 (3)	0.0060 (4)	0.0432 (16)	
C10	−0.1786 (7)	0.2886 (3)	−0.0478 (4)	0.0425 (16)	
C11	−0.0862 (6)	0.2318 (3)	−0.0616 (3)	0.0396 (14)	
C12	−0.1354 (7)	0.1741 (3)	−0.1161 (4)	0.0469 (17)	
H12	−0.0758	0.1362	−0.1279	0.056*	
C13	−0.2704 (8)	0.1716 (4)	−0.1532 (4)	0.0553 (19)	
H13	−0.3008	0.1324	−0.1893	0.066*	
C14	−0.3594 (8)	0.2273 (4)	−0.1365 (4)	0.065 (2)	
H14	−0.4507	0.2250	−0.1602	0.078*	
C15	−0.3145 (7)	0.2865 (4)	−0.0848 (4)	0.0568 (19)	
H15	−0.3745	0.3248	−0.0748	0.068*	
C16	0.2238 (7)	0.1046 (3)	−0.0807 (4)	0.0475 (17)	
C17	0.2191 (8)	0.1467 (4)	−0.1529 (4)	0.064 (2)	
H17	0.1692	0.1915	−0.1591	0.077*	
C18	0.2872 (10)	0.1225 (5)	−0.2148 (5)	0.086 (3)	
H18	0.2808	0.1499	−0.2636	0.103*	
C19	0.3657 (10)	0.0572 (6)	−0.2046 (7)	0.098 (3)	
H19	0.4180	0.0436	−0.2449	0.118*	
C20	0.3677 (10)	0.0138 (5)	−0.1382 (6)	0.093 (3)	
H20	0.4175	−0.0311	−0.1342	0.111*	
C21	0.2959 (8)	0.0346 (4)	−0.0741 (5)	0.062 (2)	
C22	0.2865 (9)	−0.0103 (4)	−0.0068 (6)	0.084 (3)	
H22	0.3361	−0.0551	0.0004	0.100*	
C23	0.2056 (8)	0.0103 (4)	0.0493 (5)	0.070 (2)	
H23	0.1929	−0.0226	0.0916	0.084*	
C24	0.1409 (7)	0.0813 (3)	0.0435 (4)	0.0439 (16)	
C25	0.0577 (7)	0.1094 (3)	0.1027 (4)	0.0447 (17)	
C26	0.0204 (6)	0.1851 (3)	0.0943 (4)	0.0383 (15)	
C27	−0.0564 (6)	0.2134 (4)	0.1515 (4)	0.0507 (17)	
H27	−0.0838	0.2638	0.1477	0.061*	
C28	−0.0932 (7)	0.1694 (5)	0.2134 (4)	0.060 (2)	
H28	−0.1419	0.1908	0.2519	0.072*	
C29	−0.0586 (8)	0.0943 (5)	0.2190 (5)	0.065 (2)	
H29	−0.0866	0.0643	0.2600	0.078*	
C30	0.0164 (8)	0.0637 (4)	0.1646 (4)	0.061 (2)	
H30	0.0404	0.0127	0.1682	0.073*	
C31	0.4148 (7)	0.2258 (3)	0.0510 (4)	0.0507 (17)	
H31	0.4105	0.2314	−0.0056	0.061*	
C32	0.5380 (7)	0.2085 (4)	0.0979 (5)	0.062 (2)	
H32	0.6156	0.2022	0.0729	0.075*	
C33	0.5461 (8)	0.2007 (4)	0.1814 (5)	0.068 (2)	
H33	0.6291	0.1894	0.2136	0.082*	
C34	0.4306 (7)	0.2098 (4)	0.2167 (4)	0.0548 (18)	
H34	0.4329	0.2039	0.2732	0.066*	
C35	0.3112 (6)	0.2276 (3)	0.1669 (4)	0.0439 (15)	
H35	0.2335	0.2352	0.1915	0.053*	

### Atomic displacement parameters (Å^2^)

**Table:**

	*U*^11^	*U*^22^	*U*^33^	*U*^12^	*U*^13^	*U*^23^
Ir1	0.04334 (15)	0.03234 (13)	0.02941 (13)	−0.00102 (12)	0.00295 (9)	0.00188 (11)
Cl1	0.0700 (12)	0.0461 (9)	0.0389 (9)	−0.0098 (8)	0.0114 (8)	0.0051 (7)
N1	0.055 (4)	0.035 (3)	0.031 (3)	0.003 (2)	0.002 (3)	0.005 (2)
N2	0.045 (3)	0.037 (3)	0.035 (3)	0.000 (2)	0.004 (2)	−0.001 (2)
N3	0.038 (3)	0.039 (3)	0.036 (3)	0.002 (2)	0.002 (2)	−0.001 (2)
O1	0.244 (5)	0.245 (5)	0.245 (5)	0.0004 (10)	0.0366 (13)	−0.0004 (10)
C1	0.063 (5)	0.034 (3)	0.034 (4)	−0.001 (3)	0.004 (3)	0.008 (3)
C2	0.073 (6)	0.038 (4)	0.053 (5)	−0.002 (3)	−0.006 (4)	0.001 (3)
C3	0.082 (6)	0.040 (4)	0.069 (5)	−0.008 (4)	−0.016 (4)	0.000 (4)
C4	0.093 (7)	0.044 (4)	0.059 (5)	−0.015 (4)	−0.010 (5)	−0.011 (4)
C5	0.106 (7)	0.037 (4)	0.044 (4)	−0.002 (4)	0.015 (4)	0.001 (3)
C6	0.068 (5)	0.038 (4)	0.035 (4)	0.001 (3)	0.012 (3)	0.004 (3)
C7	0.079 (6)	0.038 (4)	0.054 (5)	0.013 (4)	0.015 (4)	0.000 (3)
C8	0.059 (5)	0.051 (4)	0.052 (4)	0.008 (4)	0.005 (4)	0.004 (3)
C9	0.058 (5)	0.036 (3)	0.035 (4)	0.004 (3)	0.008 (3)	0.007 (3)
C10	0.053 (4)	0.038 (3)	0.033 (4)	0.000 (3)	−0.004 (3)	0.008 (3)
C11	0.049 (4)	0.038 (3)	0.030 (3)	0.001 (3)	0.000 (3)	0.009 (3)
C12	0.057 (5)	0.038 (3)	0.043 (4)	−0.004 (3)	−0.003 (3)	0.001 (3)
C13	0.073 (6)	0.048 (4)	0.041 (4)	−0.011 (4)	−0.005 (4)	−0.001 (3)
C14	0.060 (5)	0.066 (5)	0.061 (5)	−0.007 (4)	−0.017 (4)	0.004 (4)
C15	0.054 (5)	0.058 (4)	0.055 (5)	0.006 (3)	−0.004 (4)	0.004 (4)
C16	0.054 (5)	0.042 (4)	0.049 (4)	−0.004 (3)	0.013 (3)	−0.015 (3)
C17	0.095 (6)	0.049 (4)	0.053 (5)	−0.016 (4)	0.026 (4)	−0.013 (4)
C18	0.118 (8)	0.082 (6)	0.068 (6)	−0.019 (6)	0.049 (6)	−0.021 (5)
C19	0.107 (8)	0.101 (8)	0.100 (8)	−0.004 (6)	0.059 (7)	−0.029 (6)
C20	0.111 (8)	0.079 (6)	0.091 (8)	0.023 (6)	0.025 (6)	−0.034 (6)
C21	0.067 (5)	0.054 (4)	0.064 (5)	0.005 (4)	0.009 (4)	−0.017 (4)
C22	0.105 (8)	0.062 (5)	0.081 (7)	0.036 (5)	0.002 (6)	−0.007 (5)
C23	0.092 (7)	0.046 (4)	0.072 (6)	0.014 (4)	0.011 (5)	0.014 (4)
C24	0.054 (4)	0.032 (3)	0.042 (4)	−0.002 (3)	−0.003 (3)	0.002 (3)
C25	0.052 (4)	0.046 (4)	0.034 (4)	−0.008 (3)	−0.001 (3)	0.011 (3)
C26	0.035 (4)	0.048 (4)	0.030 (3)	−0.005 (3)	−0.002 (3)	0.003 (3)
C27	0.044 (4)	0.067 (4)	0.041 (4)	0.007 (3)	0.005 (3)	0.015 (3)
C28	0.035 (4)	0.101 (6)	0.043 (4)	−0.010 (4)	0.008 (3)	0.013 (4)
C29	0.058 (5)	0.087 (6)	0.050 (5)	−0.018 (4)	0.007 (4)	0.020 (4)
C30	0.076 (6)	0.056 (4)	0.048 (5)	−0.001 (4)	0.003 (4)	0.016 (4)
C31	0.053 (5)	0.052 (4)	0.047 (4)	−0.003 (3)	0.007 (3)	−0.004 (3)
C32	0.042 (5)	0.075 (5)	0.071 (6)	0.007 (4)	0.011 (4)	−0.011 (4)
C33	0.050 (5)	0.087 (6)	0.062 (5)	0.009 (4)	−0.009 (4)	−0.002 (4)
C34	0.058 (5)	0.065 (4)	0.038 (4)	−0.001 (4)	−0.003 (4)	0.003 (3)
C35	0.047 (4)	0.046 (4)	0.038 (4)	−0.002 (3)	0.005 (3)	−0.007 (3)

### Geometric parameters (Å, °)

**Table:**

Ir1—C11	1.990 (6)	C15—H15	0.9300
Ir1—C26	1.992 (6)	C16—C17	1.395 (9)
Ir1—N1	2.090 (5)	C16—C21	1.423 (9)
Ir1—N2	2.092 (5)	C17—C18	1.368 (10)
Ir1—N3	2.221 (5)	C17—H17	0.9300
Ir1—Cl1	2.5182 (16)	C18—C19	1.387 (12)
N1—C9	1.342 (7)	C18—H18	0.9300
N1—C1	1.398 (7)	C19—C20	1.331 (12)
N2—C24	1.340 (7)	C19—H19	0.9300
N2—C16	1.382 (8)	C20—C21	1.404 (11)
N3—C35	1.337 (7)	C20—H20	0.9300
N3—C31	1.351 (8)	C21—C22	1.374 (10)
C1—C6	1.404 (9)	C22—C23	1.357 (10)
C1—C2	1.406 (9)	C22—H22	0.9300
C2—C3	1.357 (8)	C23—C24	1.404 (8)
C2—H2	0.9300	C23—H23	0.9300
C3—C4	1.404 (10)	C24—C25	1.452 (9)
C3—H3	0.9300	C25—C26	1.389 (8)
C4—C5	1.341 (9)	C25—C30	1.405 (9)
C4—H4	0.9300	C26—C27	1.386 (8)
C5—C6	1.415 (9)	C27—C28	1.371 (8)
C5—H5	0.9300	C27—H27	0.9300
C6—C7	1.397 (9)	C28—C29	1.370 (9)
C7—C8	1.345 (9)	C28—H28	0.9300
C7—H7	0.9300	C29—C30	1.356 (10)
C8—C9	1.408 (8)	C29—H29	0.9300
C8—H8	0.9300	C30—H30	0.9300
C9—C10	1.460 (8)	C31—C32	1.376 (9)
C10—C15	1.392 (8)	C31—H31	0.9300
C10—C11	1.398 (8)	C32—C33	1.369 (9)
C11—C12	1.395 (8)	C32—H32	0.9300
C12—C13	1.384 (8)	C33—C34	1.366 (9)
C12—H12	0.9300	C33—H33	0.9300
C13—C14	1.375 (9)	C34—C35	1.370 (8)
C13—H13	0.9300	C34—H34	0.9300
C14—C15	1.378 (9)	C35—H35	0.9300
C14—H14	0.9300		
			
C11—Ir1—C26	87.3 (2)	C15—C14—H14	119.7
C11—Ir1—N1	80.0 (2)	C14—C15—C10	119.4 (7)
C26—Ir1—N1	93.3 (2)	C14—C15—H15	120.3
C11—Ir1—N2	94.9 (2)	C10—C15—H15	120.3
C26—Ir1—N2	80.0 (2)	N2—C16—C17	120.7 (6)
N1—Ir1—N2	171.7 (2)	N2—C16—C21	120.6 (6)
C11—Ir1—N3	173.07 (19)	C17—C16—C21	118.7 (7)
C26—Ir1—N3	87.8 (2)	C18—C17—C16	120.6 (8)
N1—Ir1—N3	105.16 (18)	C18—C17—H17	119.7
N2—Ir1—N3	79.48 (17)	C16—C17—H17	119.7
C11—Ir1—Cl1	96.67 (17)	C17—C18—C19	119.9 (9)
C26—Ir1—Cl1	174.02 (16)	C17—C18—H18	120.0
N1—Ir1—Cl1	83.02 (14)	C19—C18—H18	120.0
N2—Ir1—Cl1	104.10 (14)	C20—C19—C18	121.2 (9)
N3—Ir1—Cl1	88.68 (13)	C20—C19—H19	119.4
C9—N1—C1	118.3 (5)	C18—C19—H19	119.4
C9—N1—Ir1	113.8 (4)	C19—C20—C21	121.0 (9)
C1—N1—Ir1	127.9 (4)	C19—C20—H20	119.5
C24—N2—C16	118.8 (5)	C21—C20—H20	119.5
C24—N2—Ir1	112.8 (4)	C22—C21—C20	123.7 (8)
C16—N2—Ir1	127.7 (4)	C22—C21—C16	117.9 (7)
C35—N3—C31	117.0 (5)	C20—C21—C16	118.3 (8)
C35—N3—Ir1	121.9 (4)	C23—C22—C21	120.6 (7)
C31—N3—Ir1	120.1 (4)	C23—C22—H22	119.7
N1—C1—C6	120.4 (6)	C21—C22—H22	119.7
N1—C1—C2	120.8 (6)	C22—C23—C24	120.2 (7)
C6—C1—C2	118.8 (6)	C22—C23—H23	119.9
C3—C2—C1	120.2 (7)	C24—C23—H23	119.9
C3—C2—H2	119.9	N2—C24—C23	120.9 (7)
C1—C2—H2	119.9	N2—C24—C25	115.2 (5)
C2—C3—C4	121.5 (7)	C23—C24—C25	123.8 (6)
C2—C3—H3	119.3	C26—C25—C30	121.6 (7)
C4—C3—H3	119.3	C26—C25—C24	115.5 (6)
C5—C4—C3	119.0 (6)	C30—C25—C24	123.0 (6)
C5—C4—H4	120.5	C27—C26—C25	116.3 (6)
C3—C4—H4	120.5	C27—C26—Ir1	128.8 (5)
C4—C5—C6	121.7 (7)	C25—C26—Ir1	114.7 (5)
C4—C5—H5	119.1	C28—C27—C26	122.1 (7)
C6—C5—H5	119.1	C28—C27—H27	118.9
C7—C6—C1	119.2 (6)	C26—C27—H27	118.9
C7—C6—C5	122.0 (6)	C29—C28—C27	120.4 (7)
C1—C6—C5	118.8 (7)	C29—C28—H28	119.8
C8—C7—C6	119.9 (6)	C27—C28—H28	119.8
C8—C7—H7	120.1	C30—C29—C28	119.9 (7)
C6—C7—H7	120.1	C30—C29—H29	120.1
C7—C8—C9	120.0 (7)	C28—C29—H29	120.1
C7—C8—H8	120.0	C29—C30—C25	119.6 (7)
C9—C8—H8	120.0	C29—C30—H30	120.2
N1—C9—C8	122.1 (6)	C25—C30—H30	120.2
N1—C9—C10	115.0 (5)	N3—C31—C32	121.7 (6)
C8—C9—C10	122.9 (6)	N3—C31—H31	119.2
C15—C10—C11	121.5 (6)	C32—C31—H31	119.2
C15—C10—C9	123.3 (6)	C33—C32—C31	119.9 (7)
C11—C10—C9	115.2 (5)	C33—C32—H32	120.1
C12—C11—C10	117.0 (6)	C31—C32—H32	120.1
C12—C11—Ir1	127.9 (5)	C34—C33—C32	119.0 (7)
C10—C11—Ir1	114.9 (4)	C34—C33—H33	120.5
C13—C12—C11	121.9 (6)	C32—C33—H33	120.5
C13—C12—H12	119.1	C33—C34—C35	118.4 (7)
C11—C12—H12	119.1	C33—C34—H34	120.8
C14—C13—C12	119.6 (6)	C35—C34—H34	120.8
C14—C13—H13	120.2	N3—C35—C34	124.0 (6)
C12—C13—H13	120.2	N3—C35—H35	118.0
C13—C14—C15	120.6 (7)	C34—C35—H35	118.0
C13—C14—H14	119.7		
			
C11—Ir1—N1—C9	9.3 (4)	Cl1—Ir1—C11—C10	−90.5 (4)
C26—Ir1—N1—C9	−77.3 (4)	C10—C11—C12—C13	−1.9 (9)
N3—Ir1—N1—C9	−165.9 (4)	Ir1—C11—C12—C13	173.5 (5)
Cl1—Ir1—N1—C9	107.4 (4)	C11—C12—C13—C14	0.1 (10)
C11—Ir1—N1—C1	−173.1 (5)	C12—C13—C14—C15	1.7 (10)
C26—Ir1—N1—C1	100.3 (5)	C13—C14—C15—C10	−1.7 (11)
N3—Ir1—N1—C1	11.7 (5)	C11—C10—C15—C14	−0.1 (10)
Cl1—Ir1—N1—C1	−75.0 (5)	C9—C10—C15—C14	178.4 (6)
C11—Ir1—N2—C24	−98.7 (4)	C24—N2—C16—C17	167.2 (6)
C26—Ir1—N2—C24	−12.3 (4)	Ir1—N2—C16—C17	−22.6 (9)
N3—Ir1—N2—C24	77.2 (4)	C24—N2—C16—C21	−11.0 (9)
Cl1—Ir1—N2—C24	163.2 (4)	Ir1—N2—C16—C21	159.2 (5)
C11—Ir1—N2—C16	90.6 (5)	N2—C16—C17—C18	179.1 (7)
C26—Ir1—N2—C16	176.9 (5)	C21—C16—C17—C18	−2.6 (11)
N3—Ir1—N2—C16	−93.5 (5)	C16—C17—C18—C19	−2.4 (12)
Cl1—Ir1—N2—C16	−7.5 (5)	C17—C18—C19—C20	5.5 (15)
C26—Ir1—N3—C35	−24.1 (4)	C18—C19—C20—C21	−3.4 (16)
N1—Ir1—N3—C35	68.6 (4)	C19—C20—C21—C22	175.4 (9)
N2—Ir1—N3—C35	−104.4 (4)	C19—C20—C21—C16	−1.8 (13)
Cl1—Ir1—N3—C35	151.0 (4)	N2—C16—C21—C22	5.6 (10)
C26—Ir1—N3—C31	144.4 (5)	C17—C16—C21—C22	−172.7 (7)
N1—Ir1—N3—C31	−122.9 (4)	N2—C16—C21—C20	−177.0 (7)
N2—Ir1—N3—C31	64.1 (4)	C17—C16—C21—C20	4.7 (10)
Cl1—Ir1—N3—C31	−40.5 (4)	C20—C21—C22—C23	−174.1 (8)
C9—N1—C1—C6	4.5 (8)	C16—C21—C22—C23	3.1 (12)
Ir1—N1—C1—C6	−173.0 (4)	C21—C22—C23—C24	−6.3 (13)
C9—N1—C1—C2	−174.3 (6)	C16—N2—C24—C23	7.9 (9)
Ir1—N1—C1—C2	8.1 (8)	Ir1—N2—C24—C23	−163.8 (5)
N1—C1—C2—C3	−179.2 (6)	C16—N2—C24—C25	−174.1 (5)
C6—C1—C2—C3	1.9 (10)	Ir1—N2—C24—C25	14.2 (6)
C1—C2—C3—C4	0.0 (11)	C22—C23—C24—N2	0.7 (11)
C2—C3—C4—C5	−2.6 (11)	C22—C23—C24—C25	−177.1 (7)
C3—C4—C5—C6	3.1 (11)	N2—C24—C25—C26	−7.9 (8)
N1—C1—C6—C7	−1.4 (9)	C23—C24—C25—C26	170.0 (6)
C2—C1—C6—C7	177.5 (6)	N2—C24—C25—C30	171.9 (6)
N1—C1—C6—C5	179.7 (6)	C23—C24—C25—C30	−10.2 (10)
C2—C1—C6—C5	−1.4 (9)	C30—C25—C26—C27	1.7 (9)
C4—C5—C6—C7	−180.0 (7)	C24—C25—C26—C27	−178.5 (5)
C4—C5—C6—C1	−1.1 (10)	C30—C25—C26—Ir1	177.2 (5)
C1—C6—C7—C8	−1.1 (10)	C24—C25—C26—Ir1	−3.0 (7)
C5—C6—C7—C8	177.7 (6)	C11—Ir1—C26—C27	−81.6 (6)
C6—C7—C8—C9	0.6 (10)	N1—Ir1—C26—C27	−1.8 (6)
C1—N1—C9—C8	−5.2 (9)	N2—Ir1—C26—C27	−177.1 (6)
Ir1—N1—C9—C8	172.7 (5)	N3—Ir1—C26—C27	103.2 (5)
C1—N1—C9—C10	174.1 (5)	N1—Ir1—C26—C25	−176.8 (4)
Ir1—N1—C9—C10	−8.0 (6)	N2—Ir1—C26—C25	8.0 (4)
C7—C8—C9—N1	2.7 (10)	N3—Ir1—C26—C25	−71.7 (4)
C7—C8—C9—C10	−176.5 (6)	C25—C26—C27—C28	0.4 (9)
N1—C9—C10—C15	−177.8 (6)	Ir1—C26—C27—C28	−174.5 (5)
C8—C9—C10—C15	1.5 (10)	C26—C27—C28—C29	−2.3 (10)
N1—C9—C10—C11	0.8 (8)	C27—C28—C29—C30	2.2 (11)
C8—C9—C10—C11	−179.9 (6)	C28—C29—C30—C25	−0.2 (11)
C15—C10—C11—C12	1.9 (9)	C26—C25—C30—C29	−1.8 (10)
C9—C10—C11—C12	−176.7 (5)	C24—C25—C30—C29	178.5 (6)
C15—C10—C11—Ir1	−174.1 (5)	C35—N3—C31—C32	1.1 (9)
C9—C10—C11—Ir1	7.3 (7)	Ir1—N3—C31—C32	−167.9 (5)
C26—Ir1—C11—C12	−90.5 (6)	N3—C31—C32—C33	−0.5 (10)
N1—Ir1—C11—C12	175.7 (6)	C31—C32—C33—C34	0.5 (11)
N2—Ir1—C11—C12	−10.8 (6)	C32—C33—C34—C35	−1.1 (11)
Cl1—Ir1—C11—C12	94.1 (5)	C31—N3—C35—C34	−1.8 (9)
C26—Ir1—C11—C10	85.0 (5)	Ir1—N3—C35—C34	167.0 (5)
N1—Ir1—C11—C10	−8.8 (4)	C33—C34—C35—N3	1.9 (10)
N2—Ir1—C11—C10	164.7 (4)		
